# Gray Literature in Evaluating Effectiveness in Digital Health and Health and Welfare Technology: A Source Worth Considering

**DOI:** 10.2196/29307

**Published:** 2022-03-23

**Authors:** Sara Landerdahl Stridsberg, Matt X Richardson, Ken Redekop, Maria Ehn, Sarah Wamala Andersson

**Affiliations:** 1 University Library Mälardalen University Västerås Sweden; 2 School of Health and Welfare Mälardalen University Eskilstuna Sweden; 3 Erasmus School of Health Policy and Management Erasmus Universiteit Rotterdam Rotterdam Netherlands; 4 School of Innovation, Design, and Engineering Mälardalen University Västerås Sweden; 5 School of Health and Welfare Mälardalen University Västerås Sweden

**Keywords:** health and welfare technology, digital health, gray literature, information retrieval

## Abstract

**Background:**

The need to assess the effectiveness and value of interventions involving digital health and health and welfare technologies is becoming increasingly important due to the rapidly growing development of these technologies and their areas of application. Systematic reviews of scientific literature are a mainstay of such assessment, but publications outside the realm of traditional scientific bibliographic databases—known as gray literature—are often not included. This is a disadvantage, particularly apparent in the health and welfare technology (HWT) domain.

**Objective:**

The aim of this article is to investigate the significance of gray literature in digital health and HWT when reviewing literature. As an example, the impact of including gray literature to the result of two systematic reviews in HWT is examined.

**Methods:**

In this paper, we identify, discuss, and suggest methods for including gray literature sources when evaluating effectiveness and appropriateness for different review types related to HWT. The analysis also includes established sources, search strategies, documentation, and reporting of searches, as well as bias and credibility assessment. The differences in comparison to scientific bibliographic databases are elucidated. We describe the results, challenges, and benefits of including gray literature in 2 examples of systematic reviews of HWT.

**Results:**

In the 2 systematic reviews described in this paper, most included studies came from context-specific gray literature sources. Gray literature contributed to the overall result of the reviews and corresponded well with the reviews’ aims. The assessed risk of bias of the included studies derived from gray literature was similar to the included studies from other types of sources. However, because of less standardized publication formats, assessing and extracting data from gray literature studies were more time-consuming and compiling statistical results was not possible. The search process for gray literature required more time and the reproducibility of gray literature searches were less certain due to more unstable publication platforms.

**Conclusions:**

Gray literature is particularly relevant for digital health and HWT but searches need to be conducted systematically and reported transparently. This way gray literature can broaden the range of studies, highlight context specificity, and decrease the publication bias of reviews of effectiveness of HWT. Thus, researchers conducting systematic reviews related to HWT should consider including gray literature based on a systematic approach.

## Introduction

### Reviewing Literature in Digital Health and Health and Welfare Technology

Connected or overlapping terms are often used to describe digital interventions in health and care services, including eHealth, mobile health (mHealth), digital health, telehealth, and telemedicine [1]. The term digital health has become an established umbrella term for these [2] and implies “the use of information and communication technologies to improve human health, health care services, and wellness for both individuals and populations” [3]. The term health and welfare technology (HWT) [4], now broadly used in the Nordic countries among others, adds more detail to the digital health concept. HWT is defined as “a technology-based intervention that aims at maintaining or promoting health, well-being, quality of life, and/or increasing efficiency in the service delivery system of welfare, social, and health care services, while improving working conditions of the staff” [5]. The combination of digital health and HWT thereby encompasses broad and burgeoning interdisciplinary fields of research that may not always prioritize established scientific literature databases when publicizing results.

The Cochrane Handbook for Systematic Reviews of Interventions, considered the gold standard in conducting such reviews, recommends that “searches for studies should be as extensive as possible in order to reduce the risk of publication bias and to identify as much relevant evidence as possible” [6]. Bibliographic databases mainly index studies published in peer-reviewed journals. Depending on the question and the scope of the review, however, the proportion of relevant studies not published in scholarly journals may vary greatly. Those not indexed in bibliographic databases are frequently referred to as gray literature.

### What Is Gray Literature?

An often-cited definition of gray literature is “that which is produced on all levels of government, academics, business, and industry in print and electronic formats, but which is not controlled by commercial publishers (ie, where publishing is not the primary activity of the producing body)” [7]. Gray literature, according to this definition, is very diverse and could encompass self-published studies from research institutes, short conference abstracts, theses and dissertations, and ongoing research (trial registers), as well as government and committee reports, among others [8]. With today’s digital development and new media landscape, the related term gray data is sometimes used, including user-generated content such as blogs and social media [9].

### Is It Worth the Trouble to Include Gray Literature in Your Search?

Methodological handbooks for literature reviews promote inclusion of gray literature to increase quality and depth of reviews [6,10,11]. This can be achieved by identifying ongoing studies with useful data as well as finding additional sources for evidence above and beyond what is normally found in commercially published material. Gray literature, therefore, has the potential to reduce publication bias, which occurs when the published research is not representative of all the research that has been conducted [12].

Industry sponsors and technology developers may have different motives and timelines for work conducted in the field of digital health and HWT that may not coincide with traditional academic processes, scientific methods, or the conduct of rigorously controlled trials. In areas where the technology is developing fast, other study types than randomized controlled trials (RCT) may be preferred when evaluating interventions to allow for more rapid dissemination of results [13].

Even completed RCTs are not always published or retrievable. Al-Durra et al [14] found that 27% of all RCTs in digital health remained unpublished 5 years after completion. Industry-sponsored trials are less often published in scientific journals compared to nonsponsored or publicly funded trials, as found in a study of ClinicalTrials.gov [15]. Whether this is due to time constraints or unwillingness to widely disseminate findings, gray literature searches may reduce potential publication bias by identifying such unpublished, or ongoing, studies [16].

Moreover, rigorous and holistic assessments of evidence for interventions may not be found in bibliographic databases either. For example, publications from organizations that compile and assess evidence-based practice, such as the UK-based National Institute for Health and Care Excellence, and other health technology assessment (HTA) organizations will likely be missed if searches are restricted to bibliographic databases.

Another reason for including gray literature is that it is widely used. A recent study on nursing journals shows that gray literature accounts for about 10% of all citations [17], while another study showed that more than 10% of UK governmental publications in the field of health care are cited in the publication database Scopus [18]. Farrah and Mierzwinski-Urban [19] found that about 47% of the references on reports on novel nondrug health technologies were found in gray literature, and a review by Song et al [20] found that approximately half of all medical and health-related studies are not published in peer-reviewed journals. This can be particularly true in literature regarding new and emerging health technologies, but the reasons for not publishing studies can vary. In the Song et al [20] review, the main reason given for nonpublication was nonsubmission, where 85% of unpublished studies had not been submitted to journals due to lack of time or low priority. The next most common reason was that studies were incomplete or still ongoing. There may also be alternative incentives for publishing research in other sources than academic journals. In a study concerning the production of gray literature in the Australian public sector [21], the main reason organizations gave for publication output was to provide an evidence base to inform policy or practice, and the most effective channel for achieving this was felt to be publishing via the organization’s own website.

Gray literature can make a valuable contribution to the results in reviews of literature, although its potential relevance and contribution to the review’s results are often dependent upon the review type and its aim. Its strongest contribution may be in the conduct of scoping reviews, where it is a recommended source to search along with scientific bibliographic databases. The aim of a scoping review is to provide an overview of a field of research, quite often with a focus on providing a general picture on the available literature for a topic [22,23] or understanding the context for an intervention [9]. Gray literature suits both these purposes well. As assessment of study quality or risk of bias is generally not performed [24], gray literature may also be more easily and reliably integrated alongside scientific publications in these types of reviews.

Including unpublished studies in meta-analyses, where studies in a systematic literature review are statistically analyzed, is generally considered to improve precision in the overall result. However, it is less clear if the inclusion of gray literature influences the effect size or statistical significance [25-27]. Another advantage of including gray literature in meta-analyses reported by Halfpenny et al [26] is that they found more data in trial registers than in the published study concerning the same trial; including gray literature therefore yielded a fuller and more complete result. Integration of some types of nontrial-based gray literature alongside published studies may be more challenging, however, as requirements on the rigor and format of statistical presentations can be highly variable in comparison.

Gray literature can also strengthen systematic literature reviews without meta-analyses, especially when the focus of the review is on evaluating how new interventions function in practice—an aspect particularly relevant for digital health and HWT. This may be particularly apparent in study areas where RCTs are not standard or common such as in interdisciplinary fields [28]—a description that also fits digital health and HWT well. Such areas may have a greater proportion of lower-quality research-based evidence and a higher context specificity that is important in implementation of interventions. This is where gray literature can provide a valuable supplement to other findings [29]. Examples of this include a study by Adams et al [9] regarding public health interventions, were most or all studies were found in gray literature, demonstrating its importance in investigations of effectiveness. In a systematic review of process and implementation of participatory ergonomics, Mahood et al [22] found that the several gray literature studies included alongside peer-reviewed literature resulted in a broader view of the evidence. Similarly, a systematic review by Cooper et al [30] of public health and environmental enhancement discovered that when comparing the results retrieved from searching in several bibliographic databases with a gray literature search (foremost via web searching) the latter contributed to a greater extent to the overall synthesis of the review. A result that might be related to the interdisciplinary nature of the subject for the review, sometimes not easily found when searching more subject-specific bibliographic databases.

There are disadvantages to using gray literature, however. One is the time and resources it takes to search for it. Even though the internet has made many types of gray literature more accessible via, for example, web pages and online repositories, gray literature can still be hard to find due to its comparative deficits in metadata and indexing. The sustainability of gray literature digital domains is also a concern, with links and documents more prone to disappear than with bibliographic databases. Gray literature in many cases lacks time-saving functionality—for example, when exporting references to references management tools. It is also often less structured (eg, lacking abstracts), which makes any screening process less efficient [8,23,31].

Before undertaking a gray literature search, one should therefore consider whether the benefits outweigh the disadvantages. One example is the recommendation created by Jefferson et al [32] on whether to include the gray literature of regulatory data in Cochrane reviews concerning drugs or biologics. Key considerations include the burden of disease and number of people using or likely to use an intervention.

Because of the extra time required, Cochrane recommends limiting the use of gray literature when conducting rapid reviews and instead focusing mainly on study registers [33]. For the same reason, Cochrane recommends less frequent searches of gray literature than among publication databases when conducting and updating living reviews [34].

### Is Gray Literature a Reliable and Credible Source to Use When Reviewing Literature?

Concerns about the quality of gray literature are often raised. Literature that in many cases has not been subjected to peer review and editorial examination is often viewed as lower quality and less credible. Some types of gray literature (eg, theses and governmental reports) do, however, receive a thorough quality check before being published [23]. In the previously mentioned study of Australian gray literature [21], two-thirds of the organizations stated that they had their work externally or internally reviewed prior to publication.

**Figure 1:**
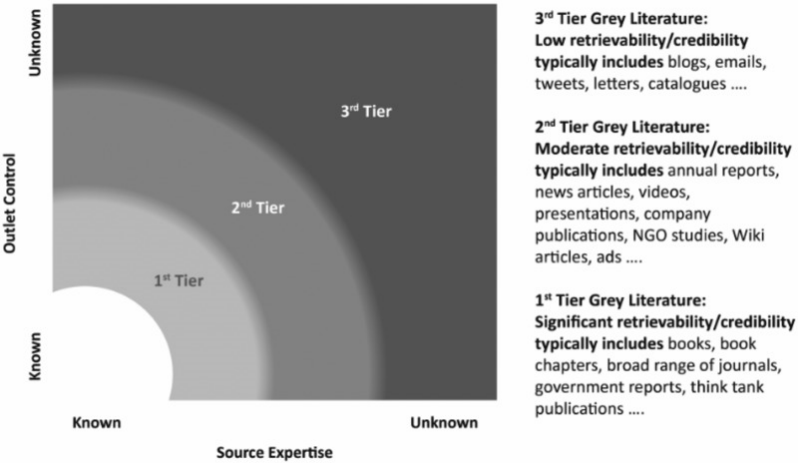
Tiers of gray literature credibility and retrievability in terms of source expertise and outlet control.

Like the evidence hierarchies within scientific literature, gray literature can be sorted according to the source’s perceived usefulness. For example, Adams et al [16] described the connection between credibility, retrievability, and outlet control in gray literature by arranging it in 3 tiers (Figure 1). The first and most credible tier consists of government reports, books, and other highly traceable publication types, followed by a second tier that includes company publications and nongovernmental organization studies. The third and final tier includes publications with little or no outlet control, such as blogs and tweets, which would rarely be included in studies of effectiveness.

When assessing bias and study quality of scientific literature, tools like the Cochrane risk-of-bias 2.0 tool for randomized trials [35] and equivalents for other types of studies are well established. The same tools can certainly be used when assessing gray literature [22,36,37]. Other forms of assessment that focus more on the value of the information may be better suited to some kinds of gray literature [9]. One approach used in several reviews [38-40] is the AACODS checklist [41], which, in line with the list’s acronym, evaluates the concepts of authority, accuracy, coverage, objectivity, date/time period, and significance of the content in a context specific to gray literature.

### What Sources of Gray Literature Are Most Relevant for Digital Health and HWT?

#### Overview

An extensive list of sources to search, including gray literature, is available in the Technical Supplement to Chapter 4 in the Cochrane Handbook for Systematic Reviews of Interventions [42]. Campbell Collaboration provides similar guidance but focuses mainly on research fields related to education [43]. Both organizations advise researchers to consult a librarian or information specialist to identify relevant sources and formulate the search strategy [42,43].

As mentioned earlier, effectiveness studies in digital health and HWT may not always be carried out as RCTs. Non-RCT studies focusing on implementation and use, for example, may more often be found in practitioner-generated gray literature as opposed to academic gray literature [44]. While academic gray literature can be found in trial registers and theses, practitioner-generated gray literature is more likely to be found in organizational reports, government documents, and evaluations accessed via web searches. For an overview of sources of gray literature, see Table 1.

**Table 1:** Overview of sources of gray literature.

Type		Example
Practitioner-generated gray literature		
	HTA^a^ organizations	Grey Matters, CADTH^b^ [45] International HTA Database [46] Evidence Search, NICE^c^ [47]
	Web search engines	Google [48] Google Scholar [49] Mednar [50] DuckDuckGo [51]
Academic gray literature		
	Dissertations, theses and academic papers	OpenDOAR^d^ [52] Open Access Theses and Dissertations [53] ProQuest Dissertations and Theses Global [54] DART Europe [55]
	Study registers	ClinicalTrials.gov [56] WHO^e^ International Clinical Trials Registry Platform [57] Finding Clinical Trials, Research Registers, and Research Results [58] JMIR Research Protocols [59]
	Conference papers	Databases indexing conference papers: Zetoc [60] Scopus [61] Web of Science [62] Embase [63] Conference journals and websites: JMIR Iproceedings [64] AMIA^f^ 2021 Virtual Annual Symposium [65]
Multidisciplinary gray literature databases		
	Multiple sources (eg, institutional repositories, digital collections, and research reports)	OpenGrey [66] OAIster [67] Bielefeld Academic Search Engine [68] GreySource Index [69]

^a^HTA: health technology assessment.

^b^CADTH: Canadian Agency for Drugs and Technologies in Health.

^c^NICE: National Institute for Health and Care Excellence.

^d^DOAR: Directory of Open Access Repositories.

^e^WHO: World Health Organization.

^f^AMIA: American Medical Informatics Association.

#### Practitioner-Generated Gray Literature

Google [48] and Google Scholar [49] are often used when searching for gray literature on the web [70]. While Google is effective for retrieving information in many superficial searches, it does not cover the deep web content and therefore needs to be supplemented with searches of databases, specific websites, and organizational repositories [12,44]. Google also uses algorithms that provide or rank search results based on previous searches [31]. The incognito option available in some browsers can therefore be useful to avoid searches from becoming biased by personal preferences or habits [71]. Another solution to avoid algorithm bias is to use alternative search engines, such as Mednar [50], which has a medical focus, or DuckDuckGo [51], a search engine that does not personalize based on previous searches.

HTA organizations evaluate digital health and HWT interventions and are thus a highly relevant source of gray literature. Grey Matters [45] from the Canadian Agency for Drugs and Technologies in Health lists HTA organizations from around the world as part of its checklist for searching gray literature.

#### Academic Gray Literature

Academic gray literature can be found in repositories for higher education, study registers, conference papers, and theses, among others. Study registers like ClinicalTrials.gov [56] and the World Health Organization (WHO) International Clinical Trials Registry Platform [57] are mandatory in Cochrane reviews for interventions to avoid publication bias [6]. This may exclude digital HWT studies, however, as they may not meet the criteria for registration in clinical trial databases due to the nature of their intervention [72]. To make protocols from ongoing studies with different study designs more accessible, initiatives such as the journal JMIR Research Protocols [59] have been established and include protocols for ongoing studies and trials in digital health and related subjects.

Research conference papers often have the highest impact in fields with rapid knowledge development of new technologies and are thus highly valued sources [73]. In other areas, or when the available evidence is poor, conference abstracts can be worth considering if there is a lack of completed studies in a field [74]. Conference papers are indexed in some publication databases—for example, the multidisciplinary databases Scopus [61] and Web of Science [62] and the medical database Embase [63]. There are also dedicated databases for conference proceedings, for example Zetoc [60], as well as journals, for example the JMIR journal Iproceedings [64]. Many recurring conferences, such as the American Medical Informatics Association Annual Symposium [65], have websites for browsing and accessing papers. Some of these more established proceedings are starting to be indexed in databases such as PubMed, however, making them less gray and easier to find in scientific bibliographic database searches. The highly referenced Institute of Electrical and Electronics Engineers (IEEE) conference papers are also indexed in IEEE’s own Xplore [75], which is considered a bibliographic database.

Academic papers can be found in national repositories for higher education such as OpenDOAR [52], a global directory of national academic repositories. International databases for dissertations and theses also exist such as Open Access Theses and Dissertations [53], ProQuest Dissertations and Theses Global [54], and Dart Europe [55], which has European content. Theses can be valuable and reliable sources of detailed knowledge on specific research topics as they disseminate results from lengthy periods of research and are subject to peer review [12]. They may also provide a more detailed background description and more extensive list of references than what is normally found in journal publications as they rarely have imposed word or page limits. Still, research that studied the impact of including theses on the result of systematic reviews from 3 Cochrane review groups found that in most reviews, their inclusion did not affect the review’s results [76]. This could explained by the fact that articles in several theses in major academic disciplines are also eventually published in scientific journals (ie, compilation theses).

#### Multidisciplinary Gray Literature Databases

There are databases dedicated to gray literature containing content from various sources—both academic and practitioner-based—and in many cases include functions for downloading references and conducting advanced searches, as in scientific bibliographic databases. A disadvantage may be that information on indexing and updating can be less transparent than for scientific bibliographic databases. There is also a greater risk that gray literature databases cease to be updated as they often lack long-term funding and organization of commercial bibliographic databases. As with scientific bibliographic databases, searching several gray literature databases may be necessary to obtain an overlapping and more complete result [12]. The subject-specific databases for gray literature can be valuable sources for locating relevant literature but are dependent on the searcher’s knowledge of the topic to identify them. Indexes for gray literature sources, such as GreySource Index [69], can therefore be valuable guides, serving as gateways to different sources of gray literature. GreySource Index is facilitated by GreyNet, an organization that provides a point of access to several repositories of gray literature.

### Is It Possible to Be Systematic When Searching Gray Literature?

There are no generally established guidelines regarding methods and search strategies for gray literature [8,12,23]. Since the extent of gray literature is vast, making a search plan prior to the search helps estimate the time and resources needed to conduct the search.

The search plan should correspond to the aim of the search and identify the sources likely to yield the most value. Three key considerations when creating a search plan are as follows:

- Which types of studies/literature are of interest?
- Which methodological guidelines need to be followed?
- Which key databases, repositories, or websites are available on the topic?

Furthermore, time period and geographic coverage should be considered [12]. A systematic gray literature search, as with any other literature search, should use a clear population, intervention, comparison, and outcome format. Searches should also be as reproducible as possible, although gray literature searches often present challenges to this. Webpages and even repositories are less stable than scientific bibliographic databases and documents and links vanish or cease to exist more regularly.

### How Should Searches for Gray Literature Be Documented?

Searches for gray literature should be documented and reported in a similar manner to searches in bibliographic databases. The aim of this documentation is to allow the search results to be reproduced by others. The Preferred Reporting Items for Systematic Reviews and Meta-Analyses (PRISMA) guidelines [77] and extension PRISMA-S [71] provide guidance on how searches should be documented and reported when conducting a systematic review. The checklist Grey Matters [45] from the Canadian Agency for Drugs and Technologies in Health also includes elements of how to document and report a gray literature search.

According to PRISMA-S [71], gray literature searches should be briefly described in the methods section of the systematic review and complemented with more detailed information in supplementary material.

### Example of Gray Literature Use in Two Systematic Reviews

#### Introduction

To illustrate how a search for gray literature can be conducted, we provide examples of methodology, benefits, and challenges taken from 2 systematic reviews we have published regarding the evidence for the use of welfare technologies: GPS alarms and digital nocturnal surveillance systems [36,37].

#### Aim of the Reviews

Both reviews aimed to find existing evidence for effects on health outcomes, welfare, and social care provision in elderly care for their respective technologies compared to standard care.

## Methods

The inclusion criteria stated that original scientific and gray literature publications regarding the specific welfare technologies produced during the period 2005-2020 in Organisation for Economic Co-operation and Development (or equivalent) country settings were included. English, French, and Nordic languages were included in order to obtain a broader base of gray literature as many public sector reports were likely produced in non-English languages. Qualitative and proof-of-concept studies, technical validations, system descriptions, reviews, and editorials were excluded for both scientific and gray literature.

The scientific database searches were conducted first and involved abstract and full-text screening steps in accordance with PRISMA guidelines. Reference and citation searches (ie, backward and forward) were also conducted for relevant publications found. The databases searched for English-language publications were Academic Search Elite (EBSCOhost), APA PsycInfo (EBSCOhost), ASSIA (Applied Social Sciences Index and Abstracts, ProQuest), CINAHL Plus (EBSCOhost), Cochrane Library [78], IBSS (International Bibliography of the Social Sciences; ProQuest), IEEE Xplore [79], PubMed [80], Scopus [61], SocINDEX (EBSCOhost), Social Services Abstracts (ProQuest), Sociological Abstracts (ProQuest), Web of Science Core Collection [62].

The search terms used in the night surveillance review were (elderly OR “older adult*” OR “older person*” OR aged [MeSH only databases]) AND (nocturnal OR “night-time” OR “nighttime” OR “night time”) AND (surveillance OR camera* OR “video monitor*” OR “in-home monitor*” OR “home monitor*” OR “safety monitor*” OR “digital monitor*” OR telemonitor* OR “remote monitor*” OR “digital camera” OR “digital sensor*” OR “monitoring system*”). Subject terms were adapted to the controlled vocabulary of each database.

In the GPS review the following search string was used: (aged OR elder* OR “older adult*” OR “older person*” OR ageing OR aging OR senior*) AND ( alarm*) AND (geofencing OR “global positioning system*” OR “positioning technolog*” OR “localization system*” OR “localization technolog*” OR “localisation system*” OR “localisation technolog*” OR “location tracking system*” OR “location tracking device*” OR “location tracking technolog*”). Also there, the search string was adapted to the subject headings of each database.

This was followed by initial searches in more established gray literature databases. These searches were conducted with the same search terms as for the scientific databases and in the English language as well. The main differences from the scientific database searches were that articles did not have to be peer reviewed, and the search strings (ie, combinations of search terms) had to be simplified in some cases as some sources’ search engines did not allow more complex strings with operator terms. In such cases, a greater number of searches consisting of fewer terms per search was required. The following resources were searched:

- Databases: Bielefeld Academic Search Engine (BASE) [68], OpenGrey [66], OAIster [67]
- HTA organization: International HTA database [46]
- Trial registers: ClinicalTrials.gov [56], WHO International Clinical Trials Registry Platform [57]
- Theses: Dart Europe [55], ProQuest Dissertations and Theses A&I [54]
- Web search engine: Google Scholar [49]

Additional searches were then performed in gray literature sources specific to the Nordic countries, where both GPS alarms and digital nocturnal surveillance systems have been implemented in social care. Due to the geographic focus, the inclusion criteria changed to studies from the Nordic countries and the searches were conducted in the Nordic languages. Repositories from higher education and websites from authorities responsible for elderly care and digitalization in health and welfare, municipalities, and research-based institutes were searched. A search was also made in Google Scholar with the respective search terms translated to the respective Nordic languages. The search string was adapted to the search functionality of the websites and search engines in a similar manner as the initial gray literature database searches.

## Results

In the GPS alarm review, 56% (9/16) of studies came from gray literature sources (8 from the Nordic sources). In the digital nocturnal surveillance systems, 60% (3/5) of publications were from gray literature, all from Nordic sources. The same bias assessment tools (Cochrane’s Risk of Bias for randomized studies and the Risk Of Bias In Nonrandomized Studies of Interventions [ROBINS-I] tool for nonrandomized studies) were used for both scientific and gray literature studies. Bias was generally found to be moderate to critical, and the result was similar between the two types of sources.

Meta-analysis or consolidation of results was not possible due to lack of rigor in statistical reporting in the gray literature publications (eg, *P* values, confidence intervals, or standard errors not calculated or reported). Instead, a narrative summary with detailed reporting of included studies’ reported results was conducted. This provided adequate transparency for the reader when drawing conclusions about the overall effect of the technologies reviewed.

## Discussion

### Lessons Learned

The inclusion of gray literature in the two reviews brought both advantages and disadvantages, but the former clearly outweighed the latter. As most included studies came from gray literature, the volume of literature to base conclusions on was considerably larger—a systematic review of only two studies would have otherwise been the reality in one case. The included gray literature studies were also highly context-specific and had excellent adherence to the reviews’ aims, perhaps even more so than the studies from the scientific database. It was, however, essentially impossible to consolidate results statistically in any meaningful way due to the lack of rigor in reporting statistics in the gray literature, which at least one journal reviewer deemed a disadvantage. It was also difficult to use the entire search strategy due to challenges with the search engines at the gray literature sources. Both the conduct of the gray literature searches and the subsequent import of found studies to the review software were far more time-consuming than in scientific publication databases; this was a far more manual process that could not use specialized applications that read the abundant metadata found in scientific databases to automate workflows. The time to review and extract data from gray literature studies was also far more time consuming due to less easily identifiable inclusion and exclusion criteria and less standardized publication formats. It is worth noting that, while the inclusion of Nordic sources and specific language searches made the findings more context-specific, it may reduce the applicability of the results elsewhere. As well, reproducing the gray literature searches may be difficult if not impossible for other researchers due to the inability to interpret specific search results and the prospective fluctuation of available documents in the searched sources. Generally, reproduction of results on effectiveness is a challenge due to complexities in implementation of HWT in specific contexts.

### Conclusion

Gray literature is worth considering when conducting many types of reviews related to HWT interventions as they can help reduce publication bias and include evidence for interventions that are not typically indexed in bibliographic databases. Including gray literature is particularly relevant for digital health and HWT, where studies tend to evaluate adoption and use using non-RCT study designs and for purposes other than primarily academic publishing. Caution needs to be taken when drawing conclusions from results in the gray literature due to a potential bias that may rise from less rigorous research design, methods, and data analyses. A risk of bias tool or checklist should be used, as with other literature. Finally, using a systematic approach when searching the gray literature is highly recommended. Planning the search and documenting it in a similar manner as when searching in bibliographic databases will save time and effort and make the search transparent and reproducible.

## Abbreviations

AACODS: authority, accuracy, coverage, objectivity, date/time period, and significance
ASSIA: Applied Social Sciences Index and Abstracts
HTA: health technology assessment
HWT: health and welfare technology
IBSS: International Bibliography of the Social Sciences
IEEE: Institute of Electrical and Electronics Engineers
mHealth: mobile health
PRISMA: Preferred Reporting Items for Systematic Reviews and Meta-Analyses
RCT: randomized controlled trial
ROBINS-I: Risk Of Bias In Nonrandomized Studies of Interventions
WHO: World Health Organization
